# MEASURING WORKPLACE LEARNING CONDITIONS FOR NURSING STAFF IN LONG-TERM GERIATRIC CARE

**DOI:** 10.1093/geroni/igac059.2857

**Published:** 2022-12-20

**Authors:** Merel (E A ) Van Lierop, Judith Meijers, Erik van Rossum, Sandra Zwakhalen

**Affiliations:** Maastricht University, Maastricht, Limburg, Netherlands; Maastricht University, Maastricht, Limburg, Netherlands; Zuyd University of Applied Sciences, School of Nursing, Heerlen, Limburg, Netherlands; Maastricht University, Maastricht, Limburg, Netherlands

## Abstract

To deal with challenges in practice such as workload and complexity in long-term care delivery, learning in practice (workplace learning) is becoming increasingly important. Nursing staff needs to take the lead and learn continuously in practice using a bottom up approach to promote workplace learning. Therefore, insight in workplace learning conditions (like work culture and learning climate) in long-term care is necessary. Based on these insights, nurses can define challenges and goals on how to stimulate workplace learning. Currently, no instruments are available that measure these conditions in the long-term nursing care setting in a snap shot. Therefore, the goal of this study was to develop a quick-scan to measure workplace learning conditions for nursing staff in long-term geriatric care. This quick-scan was developed in 3 steps: (1) investigating how to measure conditions for workplace learning, (2) selecting appropriate measurement tools to measure conditions for workplace learning, and (3) testing the feasibility of the quick-scan in practice. As a result, the final feasible quick-scan measured 11 conditions regarding workplace learning: (1) Trust: values and culture, (2) Team: support and management, (3) Colleagues: support and respect, (4) Constraints in undertaking the job, (5) Urgency, (6) Learner agency, (7) Collaboration, (8) Hybrid learning, (9) Coaching, (10) Flexibility – formal and informal learning, and (11) Assessment-as-learning. Additionally, questions were added regarding availability of sources for learning, knowledge and skills, and communication. Validated questionnaires that were included were the Culture of Care Barometer and HILL model questionnaire.

